# The cost‐effectiveness of isavuconazole compared to voriconazole, the standard of care in the treatment of patients with invasive mould diseases, prior to differential pathogen diagnosis in Spain

**DOI:** 10.1111/myc.13189

**Published:** 2020-10-30

**Authors:** José Ramón Azanza, Santiago Grau, Lourdes Vázquez, Pablo Rebollo, Carmen Peral, Alejandra López‐Ibáñez de Aldecoa, Vanessa López‐Gómez

**Affiliations:** ^1^ Clinical Pharmacology Department Clínica Universidad de Navarra Pamplona Spain; ^2^ Pharmacy Department Hospital del Mar Universitat Autònoma de Barcelona Barcelona Spain; ^3^ Hematology Department Hospital Clínico Universitario de Salamanca Salamanca Spain; ^4^ Ingress‐health Oviedo Spain; ^5^ Pharmacoeconomics Department Pfizer Madrid Spain; ^6^ Medical Department Pfizer Madrid Spain

**Keywords:** cost‐effectiveness, decision tree, invasive aspergillosis, isavuconazole, mucormycosis

## Abstract

**Background:**

Invasive mould diseases are associated with high morbidity, mortality and economic impact. Its treatment is often started prior to differential pathogen diagnosis. Isavuconazole is approved for treatment of invasive aspergillosis (IA) and invasive mucormycosis (IM) when amphotericin‐B is not indicated.

**Objectives:**

To estimate the cost‐effectiveness of isavuconazole vs voriconazole for the treatment of adult patients with possible IA prior to differential pathogen diagnosis, in Spain.

**Methods:**

A decision tree analysis was performed using the Spanish Healthcare System perspective. Among all patients with possible IA, it was considered that 7.81% actually had IM. Costs for laboratory analysis, management of adverse events, hospitalisation and drugs per patient, deaths and long‐term effects in life years (LYs) and quality‐adjusted LYs (QALYs) were considered. Efficacy data were obtained from clinical trials and utilities from the literature. Deterministic and probabilistic sensitivity analyses (PSA) were conducted.

**Results:**

In patients with possible IA and when compared to voricanozole, isavuconazole showed an incremental cost of 4758.53€, besides an incremental effectiveness of +0.49 LYs and +0.41 QALYs per patient. The Incremental Cost Effectiveness Ratio was 9622.52€ per LY gained and 11,734.79€ per QALY gained. The higher cost of isavuconazole was due to drug acquisition. Main parameters influencing results were mortality, treatment duration and hospitalisation days. The PSA results showed that isavuconazole has a probability of being cost‐effective of 67.34%, being dominant in 24.00% of cases.

**Conclusions:**

Isavuconazole is a cost‐effective treatment compared to voriconazole for patients with possible IA for a willingness to pay threshold of 25,000€ per additional QALY.

## 1 INTRODUCTION

Invasive mould diseases (IMD) are life‐threatening infections, especially in immunocompromised patients such as those with haematologic diseases, those who have been subjected to a solid organ transplantation or haematopoietic stem cell transplantation (HSCT) and in critically ill patients with leukaemia or profound neutropaenia.^1^, ^2^ IMDs are caused by fungus of the genus Aspergillus (causing invasive aspergillosis, IA) or other filamentous fungi, for example, Mucorales (causing invasive mucormycosis, IM). These infections entail an important clinical burden to individuals who are already vulnerable as they are associated with high morbidity and mortality, with mortality rates ranging from 30% to 80% for IA and up to 97% when untreated IM.^3^, ^4^ Similarly, a delay in the establishment of an adequate therapy leads to increased mortality rates in certain type of patients and significant increase in treatment duration.^5^ For instance, according to a study analysed by the FDA, a delay of 6 days in the initiation of treatment, increased mortality rates up to almost two times, from 48.6% to 82.9%.^6^ IMDs have a high economic impact as well.^1^, ^7^, ^8^ In line with available data in the website of the Spanish Ministry of Health RAE‐CMBD (Registro Atención Especializada‐ Conjunto Mínimo Básico de Datos),^9^ length of stay for IA in Spain in 2017 was 54.20 days and the mean associated costs were 18,235€; regarding IM the length of stay was 70 days and the mean costs were 24,020€.

Even though it is highly important to establish an early diagnosis for this pathology, the process is challenging because often non‐specific symptoms are present. According to the recently published update of the consensus definition of invasive fungal disease (IFD) from the EORTC and the Mycoses Study Group Education and Research Consortium, probable IFD requires the presence of a host factor, a clinical feature and mycologic evidence; cases that meet the criteria for a host factor and a clinical feature but for which mycological evidence has not been found are considered possible IMD.^10^ In contrast, obtaining a diagnosis of mucormycosis on histomorphological basis is challenging, and the most common cause for incorrect morphological diagnosis is the misidentification of Mucorales as Aspergillus spp. The application of immunohistochemistry or PCR techniques on either fresh or formalin‐fixed paraffin‐embedded tissue have been shown to be highly specific, although a variation in sensitivity has been reported.^11^ The outcome and the management of the patient depend on a prompt and correct diagnosis and usually treatment is initiated before pathogen identification.^3^ However, if the treatment turns out to be inappropriate, a delay in receiving the right treatment can increase the mortality rate, as reported by Chamilos et al where a 6‐day delay was associated with a 2‐fold increase in mortality rate.^12^

Among the therapeutic options currently available to treat IA and IM, only isavuconazole and amphotericin‐B (L‐AMB) have approved indication for both types of infections. Voriconazole and posaconazole are exclusively indicated for the treatment of IA, particularly posaconazole is only indicated when the disease is refractory to L‐AMB. According to published therapeutic guidelines, isavuconazole is recommended as a rescue therapy in IM patients, as well as posaconazole, although the recommendation for posaconazole is off‐label.^11^

The SECURE pivotal trial^13^ has shown isavuconazole to have non‐inferior efficacy and survival to voriconazole, and better safety profile, in patients with IA. On the other hand, the VITAL trial has demonstrated the efficacy of isavuconazole as primary therapy, in refractory patients and salvage, in the treatment of IM.^14^

Isavuconazole, on top of its broad spectrum, being active against Aspergillus and Mucorales, which is a clear advantage when treating patients with possible IA (thus, lacking pathogen confirmation), offers several additional advantages relative to voriconazole, that entail benefits to the patients and could carry economic consequences as well; it has lower drug‐drug interactions; therefore, it can be more safely given to patients receiving additional medications; isavuconazole does not need routinely therapeutic drug monitoring, as it is the case for voriconazole; patients receiving it show fewer adverse events, and dose adjustment is not needed for patients with mild/moderate hepatic impairment or renal impairment.^15^, ^16^

Several economic analyses of isavuconazole vs voriconazole for the treatment of IA have been conducted in different countries: in hospitalised patients with IA in the United States^17^ and in patients with possible IA in Sweden^18^ and the UK.^19^ Cost‐minimisation models of isavuconazole vs L‐AMB followed by posaconazole for the treatment of IM have been also performed in Italy,^20^ Germany^21^ and the UK.^22^ The clinical situation described in the UK cost‐effectiveness model^19^ clearly reflects the real‐world treatment approach in Spain when facing patients with possible IA, in which is necessary to start treatment before getting the differential diagnosis between IA and IM. Although isavuconazole is more expensive than voriconazole, it is effective for both IA and IM and has a better safety profile. Therefore, the objective of the present study was to conduct a health economic analysis from the Spanish National Health Service perspective (NHS), by estimating the cost‐effectiveness of isavuconazole vs voriconazole, the standard of care in Spain, for the initial treatment of IMD prior to differential diagnosis between IA and IM.

## 2 MATERIALS AND METHODS

### 2.1 Economic model

**Figure 1:**
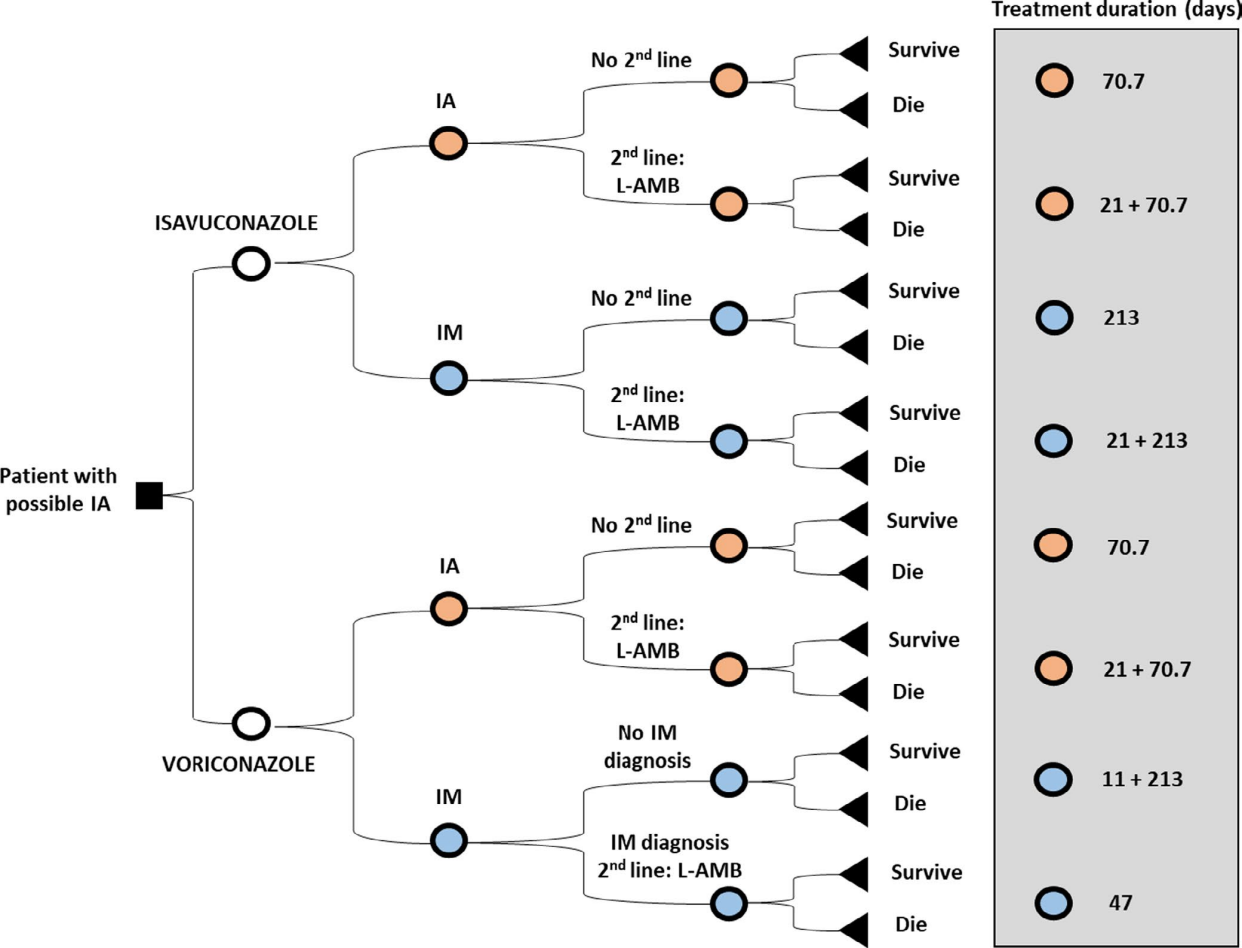
Decision tree model structure. IA, Invasive aspergillosis; IM, Mucormycosis; L‐AMB, Liposomal amphotericin‐B

A cost‐effectiveness and cost‐utility analysis, with a lifetime horizon and from the perspective of the NHS, was adapted to Spain.^19^ In order to reflect the short‐term patient pathway from initial symptoms to eventual result after antifungal treatment (resolution of infection or death), a decision tree originally developed with the collaboration of UK expert's panel was analysed by local experts (1 hospital pharmacist, 1 clinical pharmacologist and 1 haematologist, from three different autonomous communities of Spain) and considered a representative approach of Spanish patients’ pathology pathways (Figure 1).

First, the model reflects that a population of patients with ‘possible IA’ can either be treated with isavuconazole or voriconazole. Secondly, patients are subdivided into having IA or IM, regardless of a clinical confirmation. In this step, a prevalence of 7.81% was used to estimate those patients incorrectly diagnosed with possible IA but actually having IM instead (tree branches with blue dots in Figure 1). Due to a lack of specific data from Spain, this prevalence was estimated from a UK study.^23^

Then, depending on treatment response, discontinuations or diagnostic confirmation, patients may switch to a second‐line treatment or stay with the current treatment. Second‐line treatment is L‐AMB (as recommended in clinical guidelines^24^), followed by oral posaconazole or oral voriconazole.

Eventually, the model considers the possibility of having pathogen confirmation, that was set on the 11th day of treatment (which is the median time between clinical signs and IM diagnosis observed by Xhaard et al^25^ and for 50% of patients ‐ set with the experts’ panel). Regarding all IA patients and those IM patients treated with isavuconazole, pathogen confirmation does not affect the patient flow.

However, all IM patients with pathogen confirmation in the voriconazole arm will switch to second‐line treatment, as voriconazole is not indicated for the treatment of mucorales.^26^ Patients having IM and not switching are those who failed in getting a pathogen confirmation and therefore, for a short extra period of time (see treatment durations and Figure 1) they remain with inappropriate treatment.

### 2.2 Clinical inputs

#### 2.2.1 Second‐line treatment

The percentage of patients receiving second‐line treatment was calculated using data from the SECURE and VITAL trials by considering the number of patients that discontinued first‐line treatment due to no or insufficient response to treatment, adverse events or intercurrent illness.^13^, ^14^ Since no statistically differences were found between isavuconazole and voriconazole, these percentages were equally applied for both therapies (Table 1).

**Table 1:** Clinical inputs

	Pts moving to 2nd line^13^, ^14^	All‐cause mortality^6^, ^12^, ^13^	Adjusted treatment duration (d)^13^, ^14^, ^25^, ^30^, ^31^
Invasive Aspergillosis		29.07%	
1st line ISAV/ VORI	27.13%		21.00
2nd line L‐AMB + VORI/POSA	‐		70.70
Full course^c^ ISAV/VORI	‐		70.70
Mucormycosis			
1st line ISAV	14.29%	42.86%	21.00
1st line VORI	100%^a^	82.86%	11.00^b^
2nd line L‐AMB + POSA	‐	‐	213.00
Full course^c^ ISAV	‐	42.86%	213.00
Full course^c^ VORI	‐	96.20%	47.00

Abbreviations: ISAV, Isavuconazole; L‐AMB, Liposomal Amphotericin‐B; Pts, Patients; VORI, Voriconazole.

a Of those who have pathogen confirmation; ^a^

b Change to second line after pathogen confirmation; ^b^

c Patients maintaining 1st line treatment until resolution or death. ^c^

Accordingly, 27.13% of IA patients and 14.29% of IM patients treated with isavuconazole will switch to a second‐line treatment. In case of pathogen confirmation, 100% of IM patients treated with voriconazole will switch to a second‐line treatment.

#### 2.2.2 All‐cause mortality

All‐cause mortality over 84‐day from the SECURE trial^13^ (Table 1) was used to approximate mortality rate in IA patients, regardless of the initial treatment (isavuconazole or voriconazole) since no statistical differences were reported in the trial. In order to avoid double counting in the number of deaths, this mortality was also assumed for patients with IA that switch to L‐AMB.

The 84‐day all‐cause mortality in IM patients treated with isavuconazole was extracted from the VITAL study^14^ (Table 1). According to published data,^12^ an increased mortality probability due to a delay in the correct treatment was applied to IM patients treated with voriconazole that switch to L‐AMB. On the other hand, patients lacking pathogen confirmation were assumed to have the same mortality as untreated patients^6^ (Table 1).

### 2.3 Treatment regimens, dosing and duration

#### 2.3.1 Dosing

Dosing of all treatments in the model was aligned to their corresponding SmPC (refs FT) and, for L‐AMB, the FungiScope^™^ matched control study^14^ to reflect real‐world dosing (Table 2). For calculations, weight distribution of the general Spanish population was used, according to the age range of patients included in the SECURE study,^13^, ^27^ 51.10 (±16.20) years for isavuconazole and 51.20 (±15.90) for voriconazole. We then used the weighted average for 3 subgroups (35‐44 years, 45‐54 years and 55‐64 years).

**Table 2:** Treatment dosing and costs

Treatment	Price per pack (€)^32^, ^33^, ^34^, ^35^	Pack size^32^	Cost per unit (€)^32^, ^33^, ^34^, ^35^	Units per day^9^, ^13^, ^26^, ^31^	Dose per day^9^, ^13^, ^26^, ^31^
Isavuconazole					
Intravenous (Day 1 & 2)	384.00	1.00	384.00	3.00	600 mg
Intravenous (Day 3 onwards)	384.00	1.00	384.00	1.00	200 mg
Oral (Day 1 & 2)	672.00	14.00	48.00	6.00	600 mg
Oral (Day 3 onwards)	672.00	14.00	48.00	2.00	200 mg
Voriconazole					
Intravenous (Day 1)	79.99	1.00	79.99	6.00	1200 mg
Intravenous (Day 2 onwards)	79.99	1.00	79.99	4.00	800 mg
Oral (Day 1)	214.51	28.00	7.66	8.00	800 mg
Oral (Day 2 onwards)	214.51	28.00	7.66	4.00	400 mg
Liposomal Amphotericine B					
Intravenous (IA)	834.99	10.00	83.50	8.00	400 mg
Intravenous (IM)	834.99	10.00	83.50	8.00	400 mg
Posaconazole					
Oral (Day 1)	399.60	24.00	16.65	6.00	600 mg
Oral (Day 2 onwards)	399.60	24.00	16.65	3.00	300 mg

Abbreviations: IA, Invasive aspergillosis; IM, Invasive mucormycosis.

The model considers that, according to VITAL study data,^14^ 75% patients will start with IV treatment and subsequently step‐down to oral therapy, while the remaining 25% will directly start with oral therapy.

Second‐line treatment consists of IV L‐AMB followed by oral voriconazole/posaconazole at a 50%/50% ratio, except for IM patients treated with voriconazole that switch to L‐AMB: in this case, only oral posaconazole is considered. In IA, oral voriconazole was included as step‐down therapy after L‐AMB as fungal load is significantly reduced after therapy with L‐AMB and thus voriconazole is efficacious again (expert panel input).

#### 2.3.2 Duration

Treatment durations are summarised in Figure 1 and Table 1. For IA patients, it was calculated by adjusting isavuconazole treatment duration in the SECURE study (47.0 days in total: 8.1 days IV, 38.9 days oral)^28^ according to whether patients responded to, and remained on, first‐line treatment or discontinued treatment and switched to second‐line therapy. Since no statistical differences among therapies were found, a total treatment duration of 70.70 days for IA was assumed for both therapies. As previously published, the duration of second‐line therapy was assumed to be equal to the duration of first‐line therapy in patients who responded to treatment (70.70 days).^29^

Switch to second‐line treatment was assumed in the 21th treatment day (at the end of first quarter of an 84‐day therapy course). Accordingly, a patient with IA starting with voriconazole and who must switch to second‐line therapy would therefore be on treatment for 21.00 + 70.70 days. Specifically, L‐AMB treatment was assumed to last 14.50 days^30^ and oral posaconazole/voriconazole 56.20 days, calculated by subtracting 14.50 to the overall second‐line treatment duration (70.70 days).

For IM patients, total treatment duration was adjusted as for IA but using data from the VITAL study (15.5 days IV, 133.5 days oral, or 149 days for those not receiving IV). Therefore, total treatment duration for IM patients receiving isavuconazole was set as 213.00 days. The same assumptions regarding the duration of second‐line IV and step‐down therapy were made as for IA. The duration of L‐AMB was set to 27.20 days, as observed in the matched cohort from the FungiScope^™^ registry.^31^ Then, the duration of posaconazole treatment (185.80 days) was calculated subtracting L‐AMB treatment days to the total duration of second‐line therapy.

According to the model, IM patients treated with voriconazole and with pathogen confirmation switch treatment after 11.00 days,^25^ meaning patients are treated over 11.00 + 213.00 days. Whereas in patients lacking confirmation, the total treatment duration was assumed to be 47.00 days.^28^

### 2.4 Costs

Only direct costs were included in the model, namely: drug acquisition, hospitalisations, adverse events (AEs) and laboratory analysis. All costs were expressed as 2020 Euros.

#### 2.4.1 Drug acquisition costs

Acquisition costs (Table 2) were estimated using the ex‐factory price (EFP)^32^ including the corresponding mandatory deduction outlined in Royal Decree 08/2010^33^ and the reference price^34^, ^35^ established or the lowest EFP among the available.

#### 2.4.2 Hospitalisations

The cost per hospital day used in the model was 568.48€. This was calculated from Spanish health system reference costs as the mean of the latest published prices for hospital stay in every autonomous community.^36^

Length of hospitalisation stay for IA patients was extracted from the SECURE study.^13^, ^28^ Since no statically differences were found between isavuconazole and voriconazole, isavuconazole's mean hospitalisation length stay, 18.60 days, was used for both therapies. This duration was also assumed to second‐line therapy.

IM patients with a full course treatment of either isavuconazole or voriconazole were assumed to have the same hospital stay length as reported in the VITAL study,^14^ 19.30 days. In the second‐line treatment, according to the mean duration of IV therapy observed in the FungiScope^™^ case‐control study,^31^ it was assumed that patients remain hospitalised during the IV treatment, which was previously set as 27.20 days (see treatment durations).

For IM patients treated with voriconazole but switching because of pathogen confirmation, patients were assumed to be hospitalised for the entire course of their treatment prior to switching (11 days) plus the second‐line treatment hospitalisation previously detailed (27.20 days).

#### 2.4.3 Adverse events

The model includes adverse events reported in the SECURE study^15^ for which statistically significant differences between isavuconazole and voriconazole treatments were found (see Table 3). For the treatment with L‐AMB, unlike the UK original model, nephrotoxicity^37^ (associated with 9 days of additional hospital treatment^29^) and hypokalaemia^38^ were also considered. All the costs of adverse events were calculated from Spanish health system reference costs as the mean of the latest published prices for related specialist outpatient visit in autonomous communities.^36^ Due to a lack of specific information, hypokalaemia cost was assumed to correspond to a nephrology specialist visit cost (206€).^36^ Importantly, the model adjusted the average calculated AE costs by the duration of treatment for every patient subgroup.

**Table 3:** Costs included in the model

System organ class	Adverse event	Incidence of adverse event^15^		Cost (€) per event^36^
		Isavuconazole	Voriconazole	
Eye disorders	Visual impairment	0.40%	5.80%	52.00
	Reduced visual acuity	0.00%	1.50%	52.00
Hepatobiliary disorders	Hyperbilirubinaemia	0.40%	2.30%	133.58
	Abnormal hepatic function	0.80%	3.50%	133.58
	Hepatic failure	0.00%	1.20%	133.58
	Jaundice	0.00%	0.80%	133.58
	Cholestasis	0.00%	1.20%	133.58
Laboratory Tests	Increased GGT	2.30%	5.40%	157.82
	Increased ALP	1.90%	4.20%	157.82
	Increased AST	1.90%	4.20%	157.82
	Increased ALT	1.60%	4.20%	157.82
	QT prolonged	0.40%	3.10%	157.82
Psychiatric disorders	Hallucination	0.40%	4.20%	156.35
	Visual hallucination	0.00%	3.50%	156.35
Respiratory. thoracic and mediastinal disorders	Dyspnoea	3.10%	0.80%	127.51

		Liposomal Amphotericine B		
Renal disorders	Nephrotoxicity^37^	19.50%		740.96
Metabolism and nutritional disorder	Hipokalaemia^38^	53.80%		206.00

Laboratory Analysis^13^, ^14^, ^24^, ^26^, ^38^	Nº per week	Pathology		Unitary cost €^36^
Liver function test	2.00	IA & IM		14.78
Therapeutic drug monitoring of antifungal	1.00	IA & IM		107.98
Serum creatinine^a^	2.00	IM		1.80

Other costs			Unitary cost €^36^	Unitary cost €^36^
Hospitalisation day^36^				568.48

Abbreviations: IA, Invasive aspergillosis; IM, Mucormycosis.

a Only for patients treated with L‐AMB. ^a^

#### 2.4.4 Laboratory tests

Laboratory monitorisation tests were estimated taking into account length of treatment, adverse events and precautions included in the summary of product characteristics of each drug^26^ (Table 3). Liver function test was performed twice a week in all treatment groups and both for IA and IM.^13^, ^14^, ^24^, ^26^ Same applied for therapeutic drug monitoring (TDM) which was performed once a week.^39^ In the sensitivity analysis, a scenario where 10.00% of isavuconazole's patients would be monitored was analysed.^39^ Finally, for those patients having IM and treated with L‐AMB the model includes serum creatinine tests twice a week.^13^, ^14^, ^24^, ^26^ All the costs of laboratory tests were calculated from Spanish health system reference costs as the mean of the latest published prices for related test in autonomous communities.^36^

### 2.5 Utility and life expectancy

Utility and life expectancy were defined using the most frequent underlying condition of IMD in the SECURE and VITAL trials, which is acute myeloid leukaemia. Therefore, utility was assumed to be 0.82, as reported by Leunis A et al^40^ and a life expectancy of 17.00 years reported by Bower et al^41^ was used. This figure was then discounted using a 3.00% rate^42^ and the present value factor sum method resulting in an average discounted life expectancy of 13.56 years.

### 2.6 Cost‐effectiveness outcomes

Results are presented through the main cost‐effectiveness metric which is the incremental cost‐effectiveness and cost‐utility ratio (ICER/ICUR). To calculate it, both long‐term effects in life years (LYs) and quality‐adjusted life years (QALYs) were reported.

Costs and outcomes were discounted using a 3.00% discount rate.^42^ Results of the analysis were checked using a willingness to pay (WTP) threshold of 25,000 Euros per additional QALY gained.^43^ Thus, the evaluated strategy is considered cost‐effective when the ICUR < 25,000 €/QALY.

### 2.7 Sensitivity analysis

Sensitivity analyses were performed in order to evaluate the robustness of the model and analyse the uncertainty around the parameter's estimates and assumptions.

#### 2.7.1 Deterministic sensitivity analysis

A deterministic sensitivity analysis was conducted to analyse the following parameters: costs, mortality, quality of life, epidemiology and life expectancy, treatment, clinical practice, laboratory analysis and hospitalisation. In the analysis upper and lower values for parameters bounded between 0 and 1 were limited between their bounding figures. For each variable, upper and lower bounds of +20% and –20%, respectively, were assessed.

#### 2.7.2 Probabilistic sensitivity analysis

A probability distribution was defined for each parameter according to the nature of the data: parameters bounded by 0 and 1, such as percentages and health utilities, were given a beta distribution; parameters with positive figures and bounded at 0 (i.e. costs) were given a gamma distribution. The model was run repeatedly 10,000 times, taking each time a randomly selected value for each of the different inputs from its respective probability distribution (Monte Carlo simulations). Mean costs and mean QALYs were calculated using these values and results were subsequently summarised.^44^

The authors confirm that the ethical policies of the journal, as noted on the journal's author guidelines page, have been adhered to. No ethical approval was required as the research in this article related to an economic model based on data from published literature.

## 3 RESULTS

### 3.1 Base case

In the situation considered in this economic model, which is the treatment of IMD prior to differential diagnosis between IA and IM, at the point of treatment initiation, isavuconazole delivered 0.49 more LYs and 0.41 more QALYs per patient than voriconazole at an incremental cost of 4,759.53€. The ICER and ICUR were 9,622.52€/LY gained and 11,734.79€/QALY respectively (Table 4). The higher cost of isavuconazole is mainly due to drug acquisition costs since the rest of direct costs considered (management of adverse events, laboratory tests and hospitalisation days) are reduced when using isavuconazole in comparison to voriconazole.

**Table 4:** Base case analysis results

	Isavuconazole			Voriconazole			Difference
	IA	IM	Combined	IA	IM	Combined	
Costs (€)							€
Drug	10, 824.92	2,058.46	12, 883.37	6,352.79	1,291.34	7,644.14	5,239.24
Hospital	15, 841.83	1,240.52	17, 082.35	15, 841.83	1,277.27	17, 119.10	−36.74
AEs	374.45	17.63	392.08	422.71	58.14	480.85	−88.77
Laboratory analysis	291.03	55.20	346.24	673.82	26.61	700.43	−354.20
Total costs	27, 332.24	3,371.80	30, 704.04	23, 291.15	2,653.36	25, 944.52	4,759.53
Effects							
AEs (%)	33.14	2.10	35.25	69.84	5.08	74.91	−39.67
Deaths (%)	26.80	3.35	30.15	26.80	7.00	33.79	−3.65
LY	8.87	0.61	9.47	8.87	0.11	8.98	0.49
QALY	7.27	0.50	7.77	7.27	0.09	7.36	0.41
ICER per LY gained (€/LY)							9,622.52
ICUR per QALY gained (€/QALY)							11, 734.79

Abbreviations: AEs, Adverse events; IA, Invasive aspergillosis; ICER, Incremental cost‐effectiveness ratio; ICUR, Incremental cost‐utility ratio; IM, Mucormycosis; LYs, Life year gains; QALY, Quality‐adjusted life years.

Regarding LYs and QALYs, the differences between these two comparators were mostly due to the different effects of the treatments in patients with IM (higher mortality rates among patients with IM treated with voriconazole compared with those treated with isavuconazole). Importantly, isavuconazole was associated with a significant reduction of 10.79% deaths and 52.95% adverse events.

### 3.2 Deterministic sensitivity analysis

**Figure 2:**
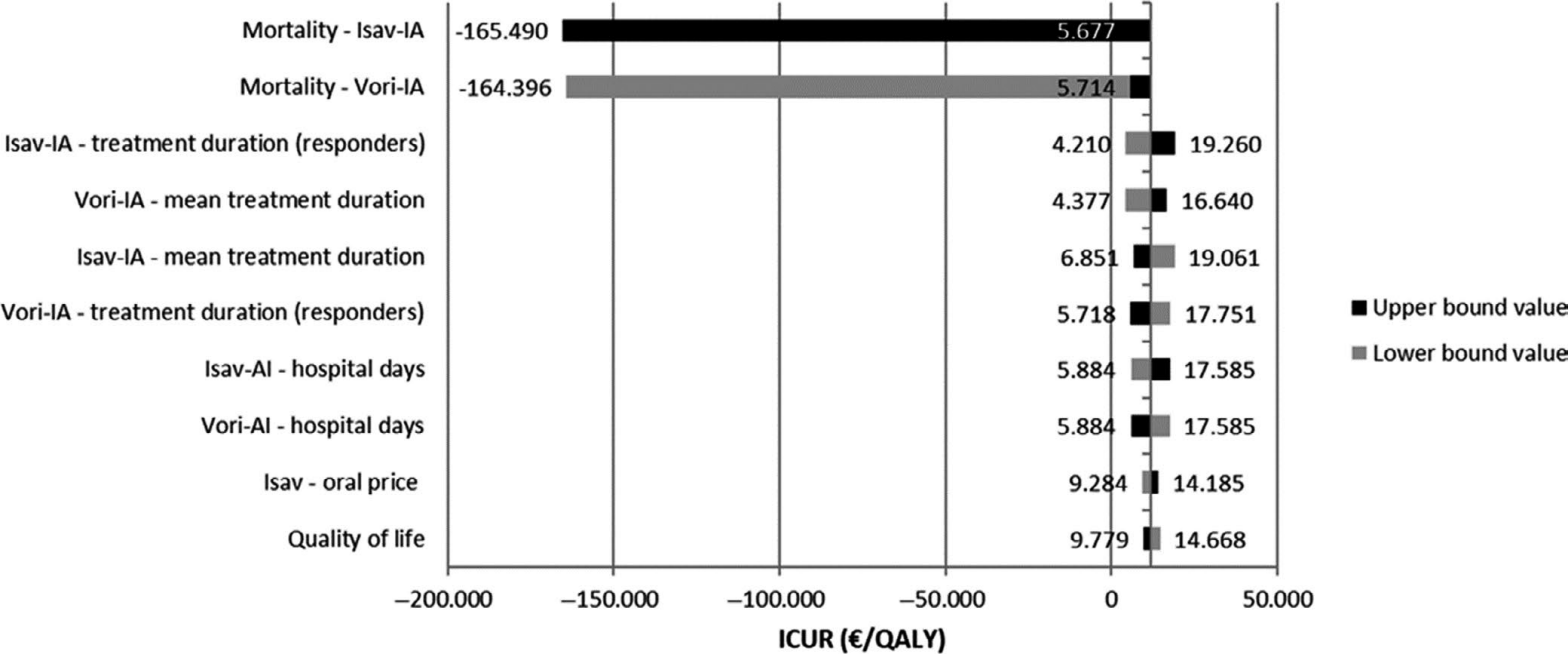
Deterministic sensitivity analysis. IA, Invasive Aspergillosis; ICUR, Incremental cost‐utility ratio; IM, Mucormycosis; Isa, Isavuconazole; QALY, Quality‐adjusted life years; Vori, Voriconazole

Values for 122 parameters were tested in the deterministic sensitivity analysis. As shown in Figure 2, main parameters influencing results were mortality, treatment duration and number of hospitalisation days. It was seen that results were especially sensitive to a reduction in mortality of IA patients treated with isavuconazole in a 20%, or an increase of this same input for voriconazole in the same percentage; the ICER was decreased to 2,600€/QALY. Additionally, treatment length of IA patients and length of hospital stay did have also important influence in the results. An increase in the number of hospital days for isavuconazole by 20% or a decrease for voriconazole, doubled the ICERs.

### 3.3 Probabilistic sensitivity analysis

**Figure 3:**
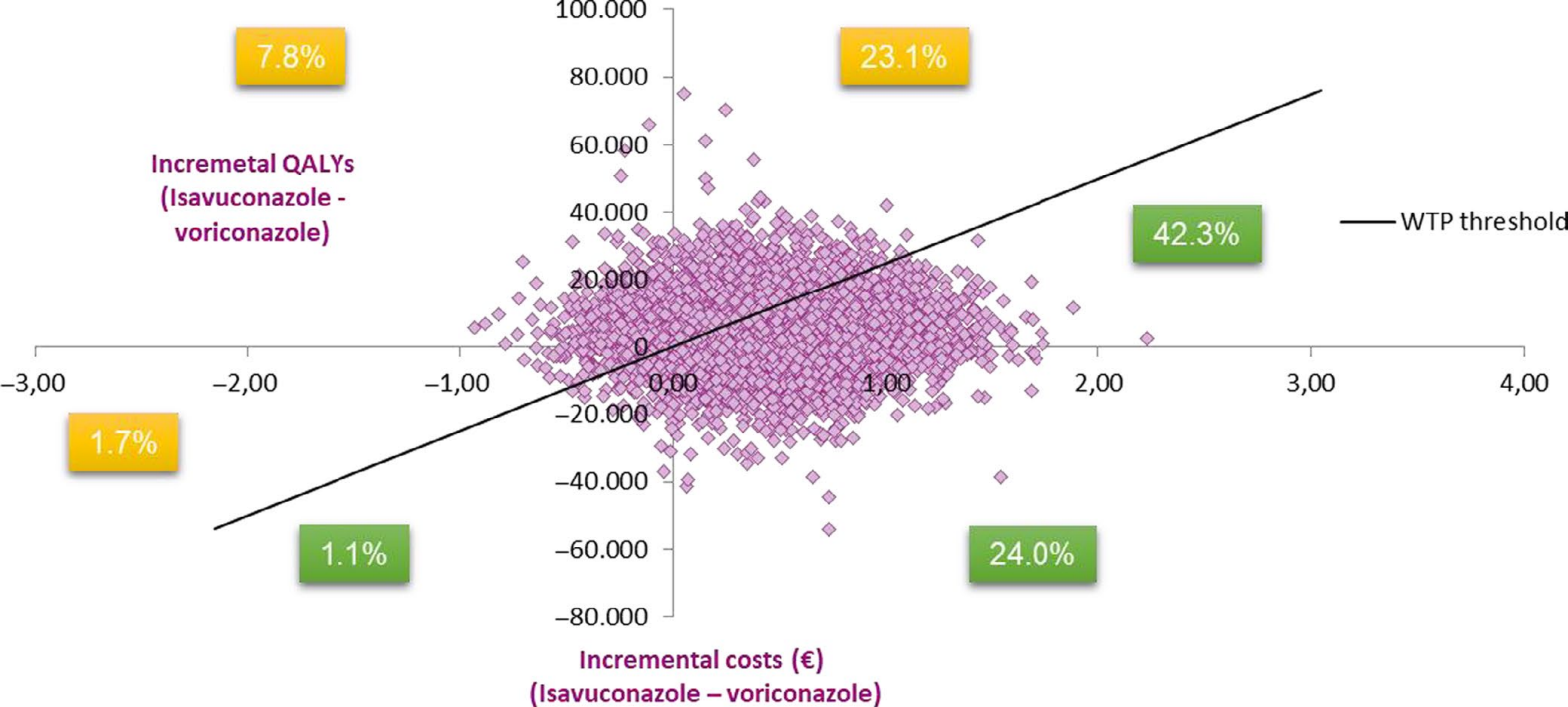
Probabilistic sensitivity analysis. WTP threshold 25 000€. QALY, Quality‐adjusted life years; WTP, Willingness to pay threshold

In the probabilistic sensitivity analysis, the average ICUR was compared with a WPT of 25, 000€ per QALY.^43^ Results showed that 65.36% of simulations (that represent the differences in costs and effects between comparators) were in the top right quadrant, indicating that isavuconazole is more efficient and more expensive in most of the cases. Besides, 24.00% of the simulations were dominant meaning more health gains and less expensive (Figure 3).

**Figure 4:**
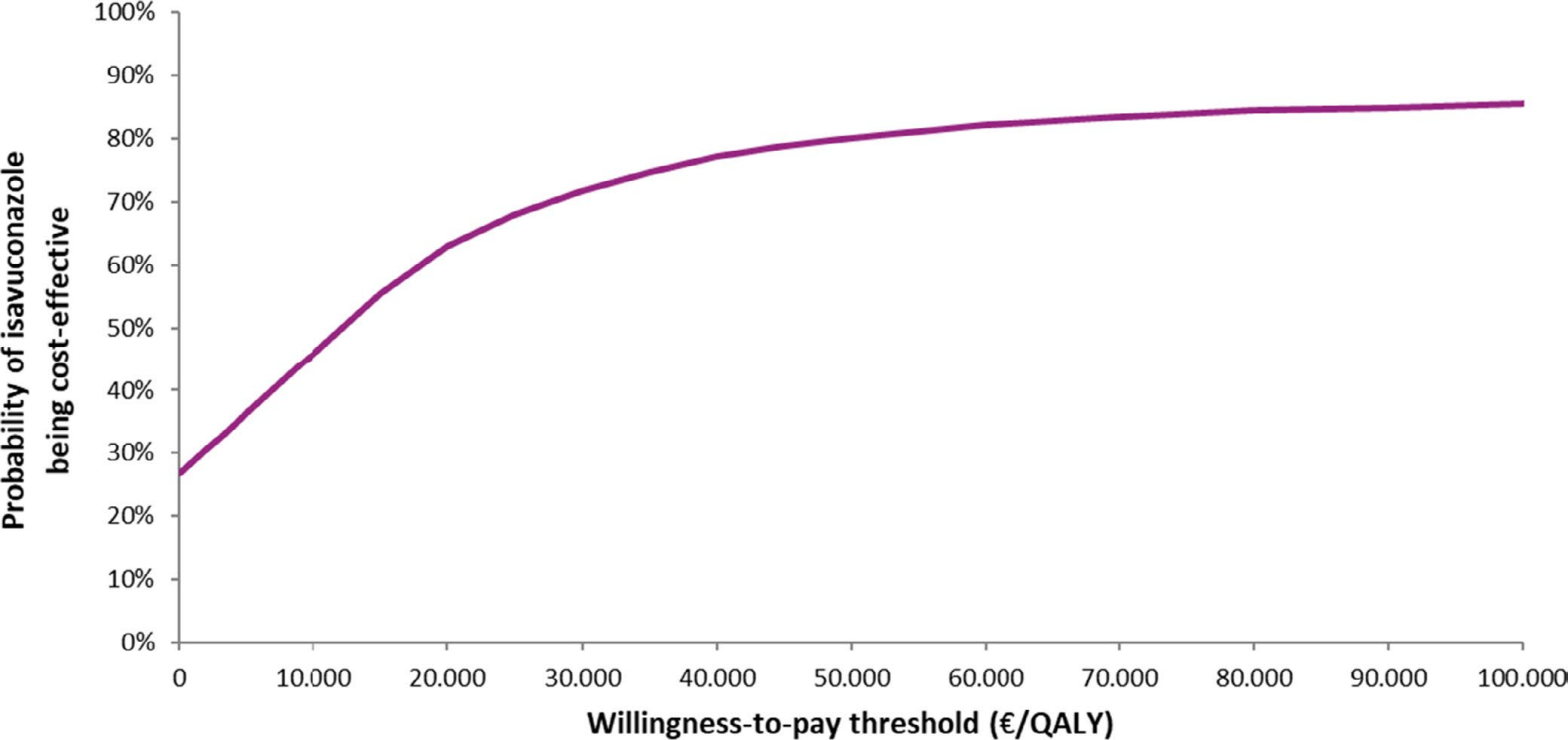
Cost‐effective acceptability curve. QALY, Quality‐adjusted life years

Data were represented in a cost‐effectiveness acceptability curve (CEAC) in order to estimate the probability of isavuconazole of being cost‐effective for a range of WPT ranging from 0 to 100,000€/QALY (Figure 4). According to these results, isavuconazole has a probability of 67.34% of being cost‐effective for a WPT of 25, 000€/QALY.

## 4 DISCUSSION

Invasive mould diseases are very severe infections that have a high morbidity and mortality, entailing high associated costs. The early differential diagnosis between IA and IM is very challenging, so in real life setting usually treatment is initiated before pathogen identification, because a delayed treatment initiation can severely influence the outcomes.^5^ Under these circumstances, isavuconazole and voriconazole are two main therapeutic options that can be chosen by the physician, taking into account both clinical and economic factors, however, only isavuconazole is effective and indicated for treating both IA and IM.^13^, ^14^, ^26^ Taking into consideration this context, this modelling study was undertaken to compare, from the Spanish NHS perspective, the cost‐effectiveness of the use of isavuconazole vs the standard of care, voriconazole, when at the time of therapy initiation, the differential diagnosis between IA and IM has not been achieved.

The base case analysis suggests that isavuconazole is cost‐effective compared with voriconazole at a WTP threshold of 25,000€/QALY. Although there is not a specific WTP threshold to be applied in health economic analyses in Spain, it has been proposed a range between 25, 000 and 60, 000 € per additional QALY gained,^43^, ^45^ meaning the analysis was made using the more conservative published threshold.

While isavuconazole is 8.23% more expensive than voriconazole, it is also associated with an increase of 0.41 QALYs and 0.49 LYs per patient. Regarding the economic cost, the main difference is the drug acquisition cost (12, 883.37€ per patient for isavuconazole vs 7,644.14€ for voriconazole), but these costs are partially compensated by a reduction in hospitalisation, laboratory analysis and adverse event costs, because of clinical outcomes improvement. Treatment with isavuconazole was also associated with a 10.79% reduction in deaths and an important reduction in adverse events (52.95%) when compared with voriconazole.

The results of the original UK model,^19^ in which this analysis was based, also favoured the use of isavuconazole with 0.39 QALYs and 0.48 more LYs per patient than voriconazole at an incremental cost of 3,228£, resulting in an ICUR of 8,242£ per additional QALY gained and an ICER of 6,759£ per additional LY gained. This was mainly due to the efficacy of isavuconazole against IA and IM, as opposed to voriconazole, which is only effective against IA. The small difference in QALYs and LYs between the original UK model and the present model is due to the different discount rate applied to the life expectancy: 3.5% in the UK model and 3% in the present one. According to the deterministic sensitivity analysis, the model was primarily sensitive to IA patients’ mortality, treatment duration and number of hospital days. The results of the Spanish model described in this paper are, therefore, well‐aligned with the previously published UK model as both are favourable to isavuconazole in the treatment of IA prior to differential pathogen diagnosis.

Other economic analysis comparing isavuconazole vs voriconazole in different settings have achieved similar results, highlighting the favourable cost‐effectiveness results of isavuconazole. A study conducted in the US comparing, from the hospital perspective, isavuconazole vs voriconazole in first‐line treatment of patients with IA,^17^ showed that isavuconazole was associated with a 7,418$ lower total cost per patient than voriconazole. Results were robust in the deterministic sensitivity analysis, with isavuconazole being dominant vs voriconazole in most cases, as well as in probabilistic sensitivity analysis where isavuconazole was cost‐effective in 82.00% of the simulations at the $50, 000 WTP threshold. A cost‐effectiveness analysis conducted from Swedish healthcare payer perspective^18^ achieved similar results of this study: isavuconazole resulted in an ICUR of 174, 890 Swedish krona (SEK) per additional quality‐adjusted life year (QALY) gained. Sensitivity analyses of this model showed that the average ICUR consistently fell below the WTP threshold of 1, 000, 000 SEK. Deterministic analysis showed that the model was primarily sensitive to mortality.

To test the uncertainty of the parameters and, therefore, the robustness of the model results, a range of sensitivity analyses were performed. In the deterministic sensitivity analysis, it was found that the results were robust under a series of alterations in the main parameters. The model was sensitive to changes in mortality of IA patients, length of treatment and hospital stay. This analysis shows the dependence of the results on the model inputs and underlying cases around the treatment of IA patients. Interestingly, all the results of the deterministic sensitivity analysis stayed below the 25, 000€/QALY threshold.

Results of the probabilistic sensitivity analysis, scattered mainly in the upper and lower right quarters, show the uncertainty about the parameters used in the model. The observed uncertainty along the horizontal axis (QALY) is mainly due to the small size of the samples. However, according to the CEAC, the probability of being cost‐effective was 67.34% with a WTP threshold of 25, 000€/QALY and increases up to 82.08% with a WTP threshold of 60, 000€/QALY, which is the range of the proposed threshold for Spain.^43^, ^45^

This study presented some limitations. In order to obtain results for the base case population, several assumptions were made regarding clinical practice, patient's underlying condition and quality of life. Firstly, a conservative prevalence of 7.81% for IM patients with possible IA diagnosis was used in the model, but higher rates have been published reporting up to 10.7%.^46^ Secondly, assuming that the patients with IA had an underlying morbidity, the application of the quality of life decrease associated to this morbidity had an impact in the results. Moreover, the model assumed that the patients surviving a fungal infection did not have any disutility due to the infection, and this might not reflect the real quality of life of these patients in the real world. Other assumptions are related to the decision tree and the numbers for mortality and treatments. The incidence of IM is low and there is a lack of data in the long‐term consequences of antimycotic treatments, thus assumptions had to be made to overcome this lack of real data in the area.

Another limitation of this cost‐effective analysis would be that the clinical parameters considered for IA patients were the same for both treatment arms (isavuconazole and voriconazole). Thus, the differences in QALYs and LYs are mainly due to the fact that voriconazole is not effective in treating IM, resulting in high mortality rates for these patients because there is a delay in effective therapy initiation (at the time the pathogen is identified); together with an improvement in IA outcomes such as reduction in mortality, laboratory costs, adverse events and hospitalisations.

## 5 CONCLUSIONS

In conclusion, isavuconazole is cost‐effective over voriconazole for the treatment of patients with possible IA. Considering the demonstrated efficacy of isavuconazole against *Aspergillus spp*. and *Mucorales spp* and its additional clinical benefits, these results strongly suggest that using isavuconazole instead of voriconazole could provide clinical and economic advantages for IA patients without pathogen identification at the moment of therapy initiation and thus being beneficial for patients and for the Spanish NHS.

## CONFLICT OF INTEREST

This analysis was sponsored by Pfizer (Spain). PR was employee of Ingress‐health Spain who received an honorarium from Pfizer (Spain) in connection with the development of this manuscript. Medical writing support was provided by PR (Ingress‐health Spain) and ALI‐A (Pfizer) and was funded by Pfizer. CP, ALI‐A and VL‐G are employees of Pfizer (Spain). JRA has received speaker and advisory honoraria from Pfizer (Spain), and SG has received speaker and advisory honoraria from Pfizer (Spain), and LV has received speaker and advisory honoraria from Pfizer (Spain).

## AUTHOR CONTRIBUTIONS

**José Ramón Azanza:** Conceptualization (equal); Methodology (equal); Supervision (lead); Validation (equal); Writing‐original draft (equal); Writing‐review & editing (equal). **Santiago Grau:** Conceptualization (equal); Methodology (equal); Validation (equal); Writing‐original draft (equal); Writing‐review & editing (equal). **Lourdes Vázquez:** Conceptualization (equal); Methodology (equal); Validation (equal); Writing‐original draft (equal); Writing‐review & editing (equal). **Pablo Rebollo:** Conceptualization (equal); Formal analysis (equal); Methodology (equal); Writing‐original draft (equal); Writing‐review & editing (equal). **Carmen Peral:** Conceptualization (equal); Funding acquisition (equal); Writing‐review & editing (equal). **Alejandra López‐Ibáñez:** Conceptualization (equal); Funding acquisition (equal); Writing‐review & editing (equal). **Vanessa López‐Gómez:** Conceptualization (equal); Funding acquisition (equal); Writing‐review & editing (equal).
